# Pathogens, reservoirs, and vectors involved in the transmission of vector-borne and zoonotic diseases in a Colombian region

**DOI:** 10.1007/s42770-023-00903-9

**Published:** 2023-02-25

**Authors:** María Cristina Carrasquilla, Mario Iván Ortiz, Daniela Amórtegui-Hernández, Sebastián García-Restrepo, Cielo León, Sergio Méndez-Cardona, Camila González

**Affiliations:** 1 Centro de Investigaciones en Microbiología y Parasitología Tropical (CIMPAT), Universidad de los Andes, Bogotá, Colombia; 2 Laboratorio de Ecología de Bosques Tropicales y Primatología (LEBTYP), Universidad de los Andes, Bogotá, Colombia

**Keywords:** Vector-borne diseases, Reservoirs, Pathogens, Vectors, Transmission cycles

## Abstract

The ecology of vector-borne diseases (VBDs) is an important system of great complexity, which involves the knowledge about the pathogens and animal species entailed in maintaining transmission cycles in a given locality, including those that act as vectors and reservoirs for the transmitted pathogens. To understand the ecology of some VBDs, we studied vectors, reservoirs, and pathogens of different VBDs, including dengue, leishmaniasis, Chagas disease, malaria, Zika, and chikungunya in the municipality of La Mesa, Cundinamarca, Colombia, a locality close to the capital, Bogotá. Vectors and mammals were sampled in urban and rural areas between May and August 2019. Molecular analyses were performed for the detection of pathogens in mammals and vectors, and of blood-meal sources in insects. Several vectors and mammals collected in this study have been involved in pathogen transmission cycles or may have a potential role in them. The findings of this study suggest that in the municipality of La Mesa, there are both vector and potential reservoir species, which are or could be implicated in the maintenance of the cycles of vector-borne diseases such as leishmaniasis and Chagas disease. Although arbovirus infections, such as dengue, are reported in the municipality, arbovirus presence was not detected. These findings highlight the importance of ongoing surveillance of vectors and associated control operations in La Mesa, of relevance to other locations where vectors and animal hosts also occur.

## Introduction

Vector-borne diseases (VBDs) are human illnesses caused by infectious agents such as parasites, viruses, and bacteria that are transmitted by arthropod species that come in close contact with humans and other animals [1]. Many of these are blood-sucking insects that ingest pathogens during a blood meal from an infected host and later transmit them into a new host. Every year, there are more than 700,000 deaths worldwide from vector-borne diseases such as malaria, dengue, human African trypanosomiasis, leishmaniasis, Chagas disease, and yellow fever, among others [1]. The burden of VBDs is higher in tropical and subtropical regions, and the poorest populations are disproportionately affected. Distribution of VBDs is determined by a complex set of environmental, biological, demographic, and social factors [1]. In Latin America and the Caribbean, the countries with the greatest risk of VBDs are Brazil and El Salvador, followed by Venezuela and Suriname; Colombia is considered in the top ten countries at greatest risk of VBDs in Latin America and the Caribbean [2].

Around 85% of the Colombian territory is located below 1600 m above sea level (m.a.s.l.), where climatic, geographic, and epidemiological conditions favor the transmission of VBDs [3]. The main VBD diseases that are transmitted in the country and subject to epidemiological surveillance are: yellow fever, malaria, dengue, leishmaniasis, Chagas disease, Zika, and chikungunya. The number of people at risk of contracting malaria is 12 million; leishmaniasis 11 million; dengue, Zika, and chikungunya 28 million; and Chagas disease 5 million [4].

Bogotá, the capital city of Colombia and the most populated city in the country is located at 2600 m.a.s.l.; it is outside the altitudinal range of suitable conditions for VBD transmission, but there is a constant movement of susceptible people between the city and surrounding towns, with active VBD transmission, during vacation periods and weekends, which increases the likelihood of outbreaks. Such is the case of the municipality of La Mesa located at 1200 m.a.s.l. This tourist municipality belongs to the Department of Cundinamarca, and it is 54 km southwest from Bogota. It has an area of 148 km^2^ and a population of around 32,000 inhabitants [5].

Between 2019 and 2021, a total of 624 VBD cases were reported in the municipality, corresponding to 599 dengue cases, three cases of severe dengue, seven chikungunya cases, nine cutaneous leishmaniasis cases, two mucocutaneous leishmaniasis cases, three Zika cases, and one Chagas disease case. In the Department of Cundinamarca, malaria cases have not been reported since 2015 [6].

In La Mesa, different phlebotomine sand flies [7], triatomine bugs [8], and *Aedes* and *Anopheles* mosquitoes have been reported [9–11]. However, an integrated study of vectors, reservoirs, and pathogens has not been performed until now. Persistence, emergence, and reemergence of the principal VBDs are the result of an intricate, intense, and dynamic interaction of different social, economic, cultural, and biological variables that generate different risk levels at transmission areas and determine the epidemiological pattern of each of these diseases in the different geographical areas of the national territory [4]. In the present study, we established an epidemiological profile of different VBDs in two localities within the municipality of La Mesa, Cundinamarca. In each site, we conducted vector and reservoir sampling, and we performed molecular detection of the pathogens that cause diseases such as dengue, leishmaniasis, Chagas disease, malaria, Zika, and chikungunya. In this way, we provide an integrated framework to study VBDs in areas at risk where constant surveillance could inform prevention strategies and anticipate outbreak occurrence.

## Materials and methods

### Study site

**Fig. 1:**
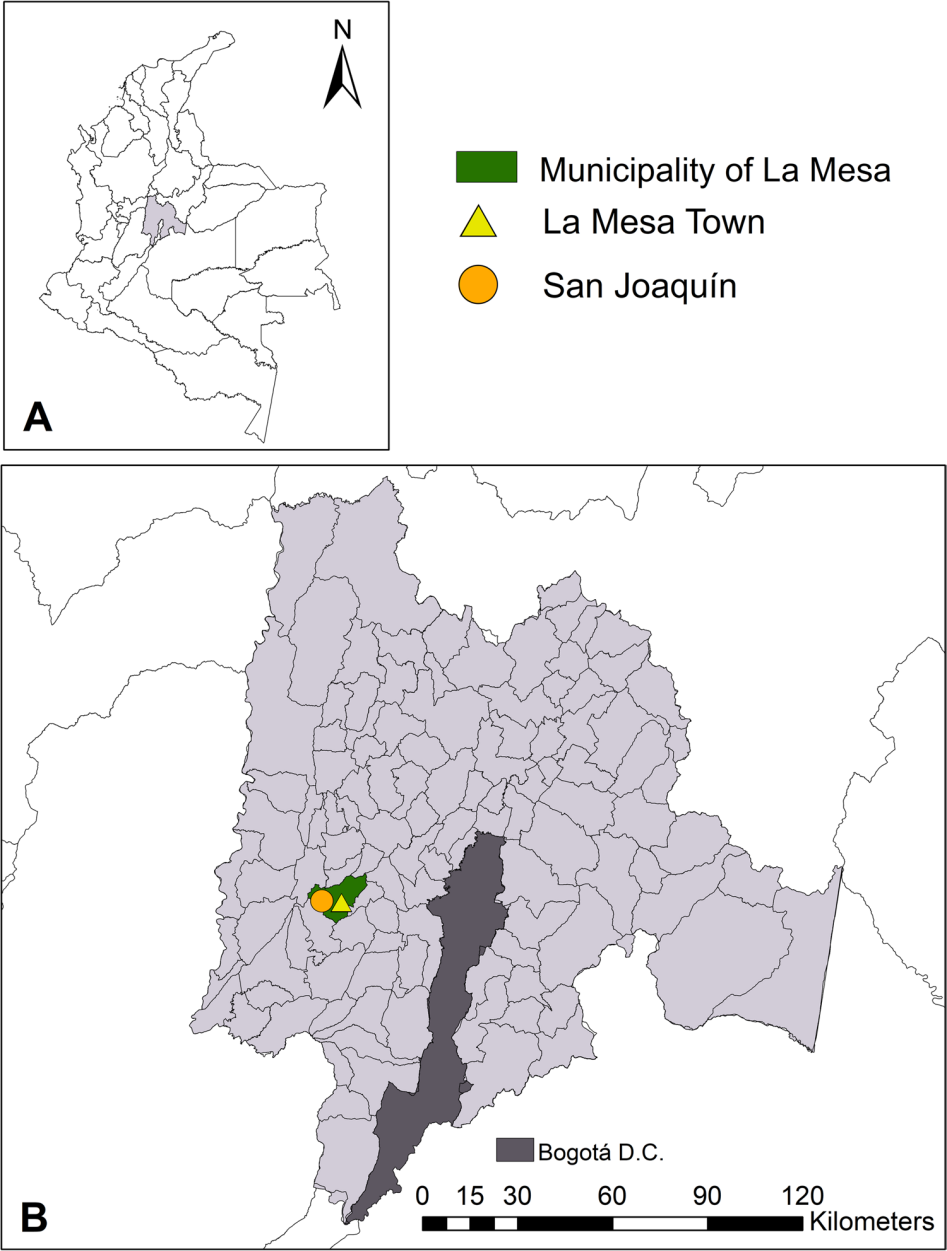
(**A**) Map of Colombia. Gray highlight shows the Department of Cundinamarca, where the investigation occurred. (**B**) Department of Cundinamarca with the municipality of La Mesa and the two localities where field work occurred

Sampling was performed in the urban and rural areas of two localities that are part of the Municipality of La Mesa (Fig. 1). Two field trips were performed. The first field trip was in May 2019 in San Joaquín Village (altitudes between 610 and 806 m.a.s.l.). During the first field trip, the average temperature was 24.84 °C, and the average relative humidity was 77.10%. The second field trip occurred between July and August 2019 in La Mesa town (altitudes between 1176 and 1416 m.a.s.l.). During the second field trip, the average temperature was 23.26 °C, and the average relative humidity was 64.32%.

### Entomological sampling

Sampling was performed in rural and urban areas. In the rural area (including areas with forest patches) in total, 24 Centers for Disease Control (CDC) light traps (John W. Hock Co., Gainesville, FL, USA) (12 per night) were installed between 18:00 and 6:00, and 24 Biogents’ mosquito traps for researchers (BG-sentinel traps) (Biogents AG, Regensburg, Germany) (12 per day) were set up over a 24-h time period. Insects were also sampled using a Prokopack aspirator (John W. Hock Co.) between 15:00 and 18:00 for 20 min at each collection site. A Shannon trap was set up for three nights between 18:00 and 22:30. In the urban zone (including the town and the surrounding areas), 24 houses were sampled (8 houses were sampled each day). In each house, a CDC trap and a BG-sentinel trap were set in the peridomicile. Also, insects were collected using a Prokopack aspirator for 20 min in the intradomicile and 20 min in the peridomicile. Live bait traps [12] were also set in available palms for collection of triatomines; in San Joaquin, 8 traps were set up, and in La Mesa, 3 traps were set.

Collection jars were maintained in a plastic bag for approximately 20 min with a cotton impregnated with ethyl acetate, for immobilizing the insects. Phlebotomine sand flies were kept in 70% ethanol. Mosquitoes were maintained in liquid nitrogen in the field and in the laboratory were stored at − 80 °C in a Revco freezer (Thermo Fisher Scientific, Waltham, MA, USA). Triatomines were preserved dry.

In the laboratory, females with blood-engorged abdomens (Culicidae and Psychodidae) were selected for blood-meal analysis and pathogen detection. Non-engorged females (Culicidae, Psychodidae, and Reduviidae) were used for pathogen detection. Identification was performed using entomological keys [9, 13–17]. For phlebotomine sand flies, all the males and some females were clarified in potassium hydroxide (KOH) and phenol [13, 14]. Because the phlebotomines were to be subjected to additional molecular analysis, identification of all females was not possible using standard practice of clearing the whole specimen. Therefore, external characteristics such as wing venation patterns and pigmentation were observed, and the head and the last segments of the abdomen were dissected and clarified in KOH and phenol to perform species identification based on the spermathecae and head characteristics [18].

### Mammal sampling

For collecting rodents and marsupials, three transects each with 25 Sherman traps and five Tomahawk traps were set up during 5 consecutive days and nights. Sherman traps were baited with peanut butter and hazelnut cream, and Tomahawk traps were baited with pineapple, banana, and yucca. Traps were baited at 17:00 h and verified at 6:00 h of the next day. Sampling of bats was carried out in forests of the rural area for 5 nights from 18:00 h to midnight, using 3 mist nets (12 m × 3 m) for each sampling site.

Only adult mammals were collected, except for pregnant and lactating females. Each individual was weighed, sexed, and morphological measurements were taken in order to make a preliminary identification using taxonomic keys [19–21]. Rodents, small marsupials (*Gracilinanus* sp.), and bats were sedated (Zoletil 50—Virbac®), and 1 ml of blood was obtained by cardiac puncture, then the sample was centrifuged at 13,000 RPM in order to obtain blood cells and plasma by separate. Thereafter, individuals were euthanized (Eutanex — INVET®), and samples of the organs (muscle, skin, heart, lung, liver, spleen, intestine, kidney, and gonads/reproductive system) were obtained. Some specimens were preserved in ethanol (70%) and others as skin, all with the skull removed. All the collected specimens were deposited in the Mammal Collection of the C.J. Marinkelle Natural History Museum, Universidad de los Andes (ANDES-M).

Blood samples from dogs and samples of blood and skin from *Didelphis marsupialis* in each location were also taken; after the extraction of the blood, the sample was centrifuged at 13,000 RPM in order to obtain blood cells and plasma separately. All samples were transported in liquid nitrogen and then stored at − 80 °C in a Revco freezer (Thermo Fisher Scientific, Waltham, MA, USA) at the CIMPAT laboratory for molecular assays. Blood cells and plasma from small mammals were stored for further assays.

### DNA extraction

DNA was extracted from individual triatomines, pools of up to ten *Anopheles* female mosquitoes, and 20 phlebotomine female sand flies from the same species, using ZR Tissue & Insect DNA Miniprep kit (Zymo REF D6016), following the manufacturer’s manual. Engorged females were processed individually.

DNA from pools of blood of up to five dogs or opossums from the same locality was extracted with Wizard® Genomic DNA Purification Kit (Promega REF A1125). If any pool was positive, individual extraction was carried out. DNA of rodents, bats, and marsupials was extracted from macerated tissue pools per individual, using the High Pure PCR Template Column Kit (Sigma-Aldrich® REF 11,796,828,001).

### RNA extraction

RNA was extracted from pools of up to 20 *Aedes aegypti* female mosquitoes using Quick RNA Viral kit (Zymo REF R1035) and from individual engorged *Aedes* mosquito using Quick DNA/RNA Viral Kit (Zymo REF D7020), following manufacturer’s manual. Organ tissues (rodents, bats, and small marsupial) from each euthanized animal was cold macerated in PBS 1 × , centrifuged, and 100 µL of the supernatant was used for RNA extraction with the Quick RNA Viral kit (Zymo REF R1035), following manufacturer’s manual.

### Pathogen detection

For *Leishmania* detection in mammal samples and phlebotomine sand flies, PCR was performed with the primers LITSR and L5.8S, which specifically amplify a fragment of 350-bp of ITS gene [22]. The positive samples by ITS were typing for hsp70 gene, following the protocol and sequencing algorithm designed by Van der Auwera et al. (2013) [23] and modifications described by Hoyos et al. (2022) [24]. Assays for detection of *Plasmodium* were performed in *Anopheles* mosquitoes. A nested PCR using ribosomal primers that amplify a fragment of 205 pb for *Plasmodium falciparum* and 117 pb for *Plasmodium vivax* was performed, following the protocol of Snounou et al. (1993) [25].

*Trypanosoma cruzi* DNA (kinetoplastid and satellite) was detected [26] in triatomines and mammal samples. Amplified fragments of 330 bp were considered positive for kinetoplastid and of 166 bp for satellite DNA. For *T. cruzi* genotyping, the amplification of the mini-exon gene was performed using PCR protocols described by Leon et al. (2019) [26]. Amplified fragments of 350 bp were considered positive for TcI and of 300 bp for TcII. We used the SL-IR region to discriminate TcI Dom and TcI Sylvatic genotypes, following PCR protocols described by Leon et al. (2015) [27].

Detection of arbovirus (Zika, dengue, and chikungunya viral RNA) was performed in *Ae. aegypti* mosquitoes and organ tissue samples using ZDC Multiplex RT-PCR Assay (Ref. 12,003,818 Bio-Rad), according to manufacturer’s instructions, as described by Carrasquilla et al. (2021) [28]. Amplification curves were evaluated by each probe, and the threshold line was placed above the background signal. Amplification curves with CT values of ≥ 37 were considered negative.

### Determination of blood-meal sources in vectors

Blood-meal analysis was performed to find out if potentially infected vectors fed on humans or other animals. Insect DNA templates were tested with primers for mammalian cytochrome B [29] and for human beta-globin gene [30]. For mammalian primer PCR, 5 μl total DNA (4 to 6 ng/µl) were mixed with the following reagents: 12.5 μl of 2 × GoTaq Green Master Mix (Promega M7123), 10 μM of forward and reverse primers to a final reaction volume of 25 μl. The thermo cycling conditions consisted of one cycle of 5 min at 95 °C, 32 cycles of 1 min at 95 °C, 1 min at 55 °C, and 1 min at 72 °C; the size of the specific PCR product was 395 bp. For human beta-globin PCR, 5 μl total DNA was mixed with the following reagents: 12.5 μl of 2 × GoTaq Green Master Mix, 10 μM of forward and reverse primers to a final reaction volume of 25 μl. The thermo cycling conditions consisted of one cycle of 5 min at 95 °C, 35 cycles of 1 min at 95 °C, 1 min at 58 °C, and 1 min at 72 °C, and finally 10 min at 72 °C; the size of the specific PCR product was 110 bp. Sanger sequencing was performed for samples that did not amplify for human-beta globin and amplified for cytochrome B gene. Sequences with an elevated identification percentage (97% or higher) in Blast were accepted as positive. Sequencing was performed in GENCORE laboratory, Universidad de los Andes, using the ABI-3500 Genetic Analyzer (Thermo Scientific. Waltham, MA, USA).

## Results

### San Joaquin Village

Regarding vectors, the mosquito species *Ae. aegypti* was captured mainly in the dwellings of the urban zone (146 females; 141 males). However, some individuals were collected in the rural area (6 females; 4 males). Dengue, Zika, and chikungunya viruses were not detected in *Ae. aegypti* females. On the other hand, the two *Anopheles* species that were collected, *An. triannulatus* and *An. neomaculipalpus*, were found only in the rural area and in the forest. Most of the sand flies (83%) were found in the rural area and in the forest. However, some were collected in the dwellings of the urban zone (17%). A triatomine bug, *Rhodnius prolixus*, was collected (fifth instar nymph), in an *Attalea* sp. palm located a few meters from a house in the urban area (Table 1). The parasites, *Plasmodium* and *Leishmania* were not detected in *Anopheles* and phlebotomine sand flies, respectively. The parasite *T. cruzi* DTU:TcI Dom was detected in the only *R. prolixus* specimen that was collected.

**Table 1:** Mosquitoes, phlebotomine sand flies, and triatomines collected in San Joaquin, Cundinamarca

Family	Species	Urban zone	Rural and forest area
Culicidae	*Aedes aegypti*	287	10
Culicidae	*Anopheles triannulatus*	0	8
Culicidae	*Anopheles neomaculipalpus*	0	11
Psychodidae	*Pintomyia ovallesi*	2	27
Psychodidae	*Pintomyia rangeliana*	2	2
Psychodidae	*Micropygomyia cayennensis*	3	15
Psychodidae	*Micropygomyia micropyga*	1	2
Psychodidae	*Micropygomyia* sp.	2	13
Psychodidae	Psychodidae (Phlebotominae) spp	2	0
Reduviidae	*Rhodnius prolixus*	1^a^	0

^a^Infected with *T. cruzi* DTU:TcI Dom

A total of 17 *Ae. aegypti* females, 1 *An. neomaculipalpus*, and 1 *Pintomyia ovallesi* were processed for blood-meal analysis. Human blood was found in 88.24% of the processed *Ae. aegypti* females and in the female mosquito *An. neomaculipalpus*. The phlebotomine sand fly species *Pi. ovallesi* that was processed was positive for chicken blood (GenBank access code: CL11_cytb OP627670). In 11.76% of the *Ae. aegypti* females, the origin of the blood-meal source was undetermined.

A total of 20 small non-volant mammals were collected, including 19 rodents and one marsupial (Table 2). In addition, four common opossums (*Didelphis marsupialis*) were sampled. *Leishmania panamensis* was detected in the tissues of a rodent of the species *Oecomys* sp. (ANDES-M 2578) collected during the sampling, as was reported in a study where the performance of the hsp70 gene sequencing was evaluated as an alternative for genotyping of *Leishmania* species [24]. On the other hand, ten leaf-nosed bats (Phyllostomidae) were captured (Table 2), and none of them was infected. Blood samples from dogs and opossums were also negative.

**Table 2:** Mammals sampled in San Joaquin, La Mesa, Cundinamarca

Order	Family	Species	*n*	Catalog number (ANDES-M)
Rodentia	Cricetidae	*Sigmodon* cf *hirsutus*	9	2565, 2568, 2569, 2572, 2573, 2574, 2580, 2581, 2582
Rodentia	Cricetidae	*Oecomys* sp.	2^a^	2567, 2578
Rodentia	Heteromyidae	*Heteromys* sp.	8	2566, 2570, 2571, 2575, 2576, 2579, 2583, 2584
Didelphimorphia	Didelphidae	*Gracilinanus* sp.	1	2577
Didelphimorphia	Didelphidae	*Didelphis marsupialis*	4	Not collected
Chiroptera	Phyllostomidae	*Artibeus lituratus*	5	2555, 2558, 2559, 2560, 2561
Chiroptera	Phyllostomidae	*Carollia perspicillata*	4	2556, 2562, 2563, 2564
Chiroptera	Phyllostomidae	*Sturnira giannae*	1	2557
Carnivora	Canidae	*Canis lupus familiaris*	30	Not collected

^a^A specimen ANDES-M 2578 infected with *Leishmania* (*Viannia*) *panamensis* [24]

### La Mesa town

The mosquito species *Ae. aegypti* was collected mainly in the urban zone (150 females; 110 males), and a female was collected in the forest. The mosquito species *An. eiseni* (1 female) and the phlebotomine sand fly species *Micropygomyia trinidadensis* (21 females and 3 males) and *Pi. ovallesi* (3 females; 1 male) were collected in the forest. The species *R. pallescens* (2 adults; 1 nymph) was collected in an *Attalea* sp. palm in the rural area (Table 3).

**Table 3:** Mosquitoes, phlebotomine sand flies, and triatomines collected in La Mesa, Cundinamarca

Family	Species	Urban zone	Rural and forest area
Culicidae	*Aedes aegypti*	260	1
Culicidae	*Anopheles eiseni*	0	1
Psychodidae	*Pintomyia ovallesi*	0	4
Psychodidae	*Micropygomyia trinidadensis*	0	24
Reduviidae	*Rhodnius pallescens*	0	3^a^

^a^A specimen infected with *T. cruzi* DTU:TcI Dom

Dengue, Zika, and chikungunya viruses were not detected in any of the *Ae. aegypti* females. Neither *Plasmodium* nor *Leishmania* parasites were detected in the *Anopheles* female mosquito or in the phlebotomine sand flies, respectively. The parasite *T. cruzi* DTU:TcI Dom was detected in one of the adult specimens of *R. pallescens*. For blood-meal analysis, 26 *Ae. aegypti* females were processed, 96.2% were positive for human blood, and in one sample the origin of the blood-meal source was undetermined. Blood-meal analysis was not performed in other vectors (sand flies, kissing bugs, and *Anopheles* mosquitoes), as they were not blood-fed.

Eight small non-volant mammals were collected, corresponding to two rodent species. Also, five common opossums (*Didelphis marsupialis*) were sampled. On the other hand, 19 leaf-nosed bats (Phyllostomidae) were captured (Table 4). In this location, pathogens were not detected in any mammal.

**Table 4:** Mammals sampled in La Mesa, Cundinamarca

Order	Family	Species	*n*	Catalog number (ANDES-M)
Rodentia	Cricetidae	*Handleyomys alfaroi*	7	2586, 2598, 2604, 2607, 2608, 2610, 2611
Rodentia	Cricetidae	*Melanomys caliginosus*	1	2609
Didelphimorphia	Didelphidae	*Didelphis marsupialis*	5	Not collected
Chiroptera	Phyllostomidae	*Anoura cadenai*	1	2591
Chiroptera	Phyllostomidae	*Anoura latidens*	1	2592
Chiroptera	Phyllostomidae	*Artibeus lituratus*	3	2588, 2595, 2600
Chiroptera	Phyllostomidae	*Carollia castanea*	2	2585, 2597
Chiroptera	Phyllostomidae	*Carollia perspicillata*	11	2587, 2590, 2593, 2594, 2596, 2599, 2601, 2602, 2603, 2605, 2606
Chiroptera	Phyllostomidae	*Glossophaga soricina*	1	2589
Carnivora	Canidae	*Canis lupus familiaris*	32	Not collected

## Discussion

The effective transmission of pathogens by vectors requires a series of factors (biological and non-biological) that have to coincide in a specific time and site [31]. Among these biological factors are the pathogens and the species involved in the maintenance of the transmission cycles in a determined area and time. In this report, we studied vectors, pathogens, and reservoirs of different vector borne diseases, including dengue, leishmaniasis, Chagas disease, malaria, Zika, and chikungunya in La Mesa, Cundinamarca.

Vector insects belonging to the Triatominae subfamily were sampled during the development of this study, particularly the species *R. prolixus* and *R. pallescens*. In Colombia, the kissing bug *R. prolixus* is the main vector of *T. cruzi,* the parasitic protist that causes Chagas disease in humans [32]. The natural habitat of *R. prolixus* are large-crown palms, particularly *Attalea butyracea* [33]. *Rhodnius prolixus* has also been collected in human dwellings, but colonization remains a matter of debate in Colombia and Venezuela because of reinfestation by silvatic populations [33, 34]. This species has been the main target of vector control programs in Colombia, whose efforts have resulted in the interruption of intradomiciliary transmission of *T. cruzi* by *R. prolixus* in 66 municipalities [35]. In the present study, one fifth instar nymph was collected in a palm tree (*Attalea* sp.), located near a house in the urban area; no specimens were found inside the house, therefore surveillance is recommended.

The other triatomine species sampled was *R. pallescens*, a sylvatic vector related to palm trees*,* and a visitor of houses located near palm trees, although without establishing intradomestic colonies [36]. This species feeds mainly on humans, marsupials, and squirrels [35, 37], and it is the main vector of Chagas disease in Panama, and it is also found in Costa Rica, Nicaragua, Belize, and Colombia [35]. In Colombia, *R. pallescens* plays an important role as a secondary vector of *T. cruzi* [37]; it has a great association with peridomestic and wild habitats, and in the future, its role in the transmission of Chagas disease may increase, thus, the monitoring of this species should be included in surveillance programs [37]. Its wide distribution and its visits to houses located near palm trees make this triatomine bug a potential problem, since it is a candidate to replace the domestic *R. prolixus*, if it is eliminated from houses, as a consequence of control campaigns [36, 37]. *Rhodnius pallescens* specimens collected in the present study were all captured in an *Attalea* sp. palm tree near a house, which corresponds to the habitat described for this species [35–37].

Regarding the infection by *T. cruzi* of the triatomine specimens collected during the development of this study, one *R. prolixus* and one *R. pallescens* were found infected with the parasite. The molecular characterization of the pathogen showed that both triatomine specimens were infected with *T. cruzi* DTU: TcI Dom. The parasite *T. cruzi* presents tremendous intraspecific genetic diversity and has been divided into seven discrete typing units (DTUs, TcI to TcVI), including a genotype associated with anthropogenic bats, called TcBat [38, 39]. These DTUs show some associations with the ecology, transmission cycle (domestic/sylvatic), and clinical manifestations of the disease to some extent [39]. TcI is the most widely distributed DTU in the Americas with a distribution that covers the southern USA to northern Argentina and Chile [27]. Although the circulation of all DTUs has been described in Colombia, TcI is the most distributed DTU in this country [38]. DTU TcI is subdivided into two genotypes associated with transmission cycles: domestic (TcI Dom) and sylvatic (TcI sylvatic) [40]. TcI Dom is characterized by an association with human infections and domestic triatomines, but it is important to mention that TcI Dom is also detected in the sylvatic cycle, but this is explained by the movement of insect vectors and synanthropic reservoirs between transmission cycles [39].

Taking into account the results obtained in this study, we can mention that in this area, it is likely that the life cycle of *T. cruzi* involves sylvatic *Rhodnius prolixus* or *R. pallescens* as vectors and the mammalian species *D. marsupialis* and *C. lupus familiaris* as possible reservoirs. Although in the present study, no mammals infected with *T. cruzi* were detected; in Colombia, the infection of the species *D. marsupialis* with this parasite has been demonstrated [41] and anti-*T.cruzi* antibodies have been found in *C. lupus familiaris* individuals, sampled in La Mesa municipality with a seroprevalence of 29.5% [42]. In La Mesa municipality, from 2012 to 2021, only three cases of Chagas disease were reported. Specifically, in the year in which the present study was carried out (2019), cases were not registered despite the fact that vectors infected with *T. cruzi* were found in the area, as shown by our results [6]. Moreover, oral transmission of Chagas disease has been identified as the mechanism involved in the development of several outbreaks in Latin America [42]. Several of these outbreaks have happened in urban places, for example, Caracas (Venezuela) and Bucaramanga (Colombia) [43, 44]. This type of transmission in urban places highlights the importance of surveillance programs in places like La Mesa, where domiciliated triatomines were not found, but *T. cruzi-*infected triatomines were detected in areas near houses.

In this study, *Leishmania* (*Viannia*) *panamensis* was detected in the rodent genus *Oecomys*, as was previously reported in a study about *Leishmania* detection using hsp70 gene [24]. The genus *Oecomys* belongs to the family Cricetidae, and its distribution includes Panama, Bolivia, Colombia, Venezuela, and Trinidad Island [45, 46]). *Leishmania* (*Viannia*) *panamensis* is recognized as an etiological agent of cutaneous leishmaniasis [47]. In Colombia, the rodent species *Oecomys trinitatis* was found positive for *L.* (*Viannia*) spp. [48]. Also, in Brazil, other trypanosomatids, specifically *T. dionisii*, have been detected in rodents of this genus [49].

Animals infected with zoonotic species of *L.* (*Viannia*) belong to the orders Carnivora, Cingulata, Chiroptera, Didelphimorphia, Lagomorpha, Pilosa, Primates, and Rodentia. This last order is the most extensively studied [50]. The presence of zoonotic species of the subgenus *L.* (*Viannia*) has been reported in 27 Rodentia species including *Rattus rattus* and *Mus musculus.* The key role of the Rodentia species as reservoirs of species of the subgenus *L.* (*Viannia*) may be due to some reasons: their proximity to humans, high prevalence values, and their abundance in ecological niches where phlebotomines are found [50].

As far as we know, the only mammal species reported infected with *L.* (*Viannia*) *panamensis* is *Choloepus hoffmani* (two toed sloth) [50]. The infection by *L.* (*Viannia*) *panamensis* of the rodent genus *Oecomys* sp*.*, collected during the development of this project, is the first record of infection by this species of *Leishmania* in this genus of rodent and the first of this species of *Leishmania* in this order of mammals.

Even though bats collected in this study were not found infected, these organisms are an important group of study for public health due to the association between them and the transmission of zoonotic diseases such as rabies, Ebola, and SARS [51]. In the Neotropics, it is estimated that half of the mammalian fauna is represented by bats, where Colombia is the country with the second highest diversity (217 species) worldwide, after Indonesia [52]. These organisms have been on earth since 52.5 million years ago and can harbor many pathogens [53]. Regarding the infection of arbovirus, bats have been reported as hosts of arbovirus of medical and veterinary importance [54]. However, the contribution of bats in the circulation of arboviruses is not clear [55]. There have been reports of serological and molecular evidence of different bat species infected with dengue virus (DENV), yellow fever virus, Zika virus, and chikungunya virus. Particularly, species found in this study such as *Carollia perspicillata*, *Artibeus lituratus*, and *Glossophaga soricina* have been found infected with arboviruses such as yellow fever and dengue [55].

The parasites of the genus *Trypanosoma* have a close association with bats, especially those belonging to the subgenus *T.* (*Schizotrypanum)*, including *T.* (S*chizotrypanum*) *cruzi* [56, 57]. Trypanosomes have been reported in more than 70 species of bats from a wide range of families and are found mainly in insectivorous species, suggesting that they can be infected by ingestion of infected arthropods [58]. Regarding other trypanosomatids, such as *Leishmania*, different species including *L. infantum*, *L. amazonensis*, *L. braziliensis*, and *L. mexicana* have been detected infecting bat species reported in this study. Particularly, *L. infantum* has been detected in *C. perspicillata*, *G. soricina*, and *Ar. lituratu*s, *L. amazonensis* in *Ar. lituratu*s, *L. braziliensis* in *G. soricina*, and *L. mexicana* in *Ar. lituratus* and *G. soricina* [50]. In Colombia, different studies have detected *T. cruzi* in several wild bat species, including species collected in this study, such as *C. perspicillata*, *Ar. lituratus*, and *G. sorcina* [44, 57, 59, 60]. Also, *Leishmania* spp. have been detected recently in bat species (*Molossus molossus* and *Phyllostomus hastatus*) collected in the country [59, 60].

This study also constitutes the first report for the area (Department of Cundinamarca) of the bat species *Sturnira giannae*, found previously in Santander, Arauca, Caquetá, Putumayo, Tolima, and Risaralda [61–64], and also the first report of *Anoura cadenai*, previously found in Valle del Cauca, Nariño, Putumayo, Huila, and Risaralda, but not reported in the eastern mountain region [65–69]. These genera are particularly relevant because for the genus *Sturnira*, serological evidence of infection by DENV and by Venezuelan Equine Encephalitis Virus (VEEV) has been found [55]. DENV is the arbovirus with more reported cases in the area, although it was not detected in the present study; regarding VEEV, outbreaks has been registered in Colombia [70], but no cases of infection by this virus has been reported in the area. Likewise, the genus *Anoura* has also been found infected, by serological evidence, with DENV and Ilheus virus [55].

In San Joaquin, we collected four phlebotomine sand flies species (*Pi. ovallesi*, *Mi. cayennensis*, *Mi. micropyga*, and *Pi. rangeliana*). The species *Mi. cayennensis* has been found naturally infected with *L. panamensis* in Northern Colombia [71]. *Pintomyia ovallesi* has been incriminated as a vector of *L. braziliensis* and *L. mexicana* [72] and *Pi. rangeliana* has been found infected with promastigotes [73]. *Micropygomyia cayennensis* feeds mainly on lizards, and it is found inside dwellings, particularly if geckos are present. In high densities, it has been found feeding on humans [74], and recent molecular analysis of blood-meal sources have detected that this species also feeds on dogs and ducks [71]. In San Joaquin, the natural cycle of *Leishmania panamensis* may involve this species; however, the role of this species in the transmission of cutaneous leishmaniasis in Colombia remains to be determined. Our findings on blood-meal analysis show that *Pi. ovallesi* feeds on chicken blood. Other studies developed in the Department of North of Santander, Colombia have shown that this species feeds frequently on humans, cows, and horses [75]. In laboratory setting, this sand fly species feeds on different blood sources, including chicken blood [76].

During the development of this study, specimens belonging to the genus *Anopheles* were captured, particularly the species *An. triannulatus* s.l., *An. neomaculipalpus*, and *An. eiseni*. Colombia has a high number of anopheline mosquito species, compared to neighboring countries and is a potential hotspot for malaria endemicity [77]. Five subgenera of *Anopheles*, with between 40 and 47 species are found in the country [9]. The species *An. neomaculipalpus*, captured in this study, is considered a secondary malaria vector in Colombia [78]. This species is widely distributed from Mexico to Argentina [78], and in Colombia, it has been collected in 27 out of 32 departments [9]. In Venezuela, it has been reported as a highly anthropophilic species [79] and has been found infected with *Pl. vivax* [78]. Although the specimens captured during this study were not found infected, in one female, human blood was detected, and this species has been found naturally infected with *Pl. falciparum* in Colombia [80, 81]. The other two species of *Anopheles* captured in La Mesa municipality (*An. eiseni* and *An. triannulatus* s.l.) are not incriminated as malaria vectors in Colombia [78, 82]. *Anopheles triannulatus* has a wide distribution in Central and South America and in Colombia; it is distributed in 27 of the 32 departments [9]. Despite its non-relevant role in malaria transmission in Colombia, *An. triannulatus* has been found naturally infected with *Pl. vivax* and *Pl. falciparum* in the country [83, 84]. In some regions of Brazil and Peru, it is considered a secondary vector [85]. In relation to *Anopheles eiseni*, it is a species of mosquito historically distributed across much of South and Central America including countries such as: Mexico, Brazil, Bolivia, Ecuador, and Colombia [78, 86]. In Colombia, this species of mosquito has a wide distribution that includes 19 out of 32 departments [9]. *Anopheles eiseni* is unlikely to be a major malaria vector, since it is not an avid blood-sucker, although successful laboratory infection with *Pl. falciparum* has been demonstrated [87]. Although mosquitoes of the *Anopheles* genus, captured and identified in this study, were not found infected with parasites of the *Plasmodium* genus, the internal migration of people in Colombia may present a high risk for the introduction of these parasites in this area, that shows a high flow of people from different regions of the country. The malaria transmission in Colombia is characterized by outbreaks, caused in part by the high internal migration [88].

Despite the fact that 296 *Ae. aegypti* females were captured in the urban zone and seven in the rural area, none was infected with DENV, chikungunya virus, or Zika virus. This fact contrasts with the number of dengue cases that have been reported in recent years in the area; between 2012 and 2021, 1881 cases were registered [6], including dengue and severe dengue. Specifically, in the year (2019) in which the present study was carried out, 218 dengue cases were reported [6]. In this study, we did not detect DENV in *Ae. aegypti* females which may be due to the low number of mosquitoes collected. Regarding chikungunya and Zika viruses, a decline in the number of reported cases has been detected after the epidemics registered in 2014–2015 and 2015–2016, respectively. Particularly, in the area, four chikungunya cases and three Zika cases were reported in 2019 [6]. Even though *Ae. aegypti* is associated with urban settings, we also report the presence of this mosquito in rural areas as was registered by Morales (1981) [89]. In this study, *Ae. aegypti* mosquitoes captured in rural areas were found in the peridomicile of isolated farms and in the forest near these farms.

In conclusion, although infection was not detected in most of the insect and mammal species identified in this study, several of these species have been involved in pathogen transmission cycles or may have a possible role in them. Transmission cycles in La Mesa involve the circulation of the parasites *Trypanosoma cruzi* TcI Dom and *Leishmania* (*Viannia*) *panamensis*; the circulation of these pathogens is consistent with the leishmaniasis and Chagas disease case reports in recent years in this locality. In contrast, dengue infection in humans is the most frequent infection by vector-borne pathogens reported in this area, but it was not possible to detect insects infected with this virus in the present study, despite the fact that the collections were made in the period of the year in which there was a peak in the number of human dengue cases in the area, particularly, 52 dengue cases were registered between May and August out of 218 reported in 2019 [6]. This lack of virus dengue detection is probably due to the low number of mosquitoes collected. However, in other studies carried out in the same location (San Joaquin, La Mesa) [11] and in Arauca, Colombia [90] and Kuala Lumpur, Malaysia [91], dengue virus was detected in the sampling between 52 and 148 *Aedes* mosquitoes. These findings show that in this municipality there are both vector and potential reservoir species, which are or could be implicated in the maintenance of the life cycles of several vector-borne diseases such as leishmaniasis and Chagas disease. These findings as well as the case reports, in this area, highlight the importance of surveillance and vector control campaigns in this area.

## Data Availability

The partial nucleotide sequence of *Gallus gallus* isolate CL11 cytb gene identified for blood meal analysis was deposited in GenBank (Access code: OP627670) (https://ncbi.nlm.nih.gov/genbank/).
